# GPT-4o and OpenAI o1 Performance on the 2024 Spanish Competitive Medical Specialty Access Examination: Cross-Sectional Quantitative Evaluation Study

**DOI:** 10.2196/75452

**Published:** 2026-01-12

**Authors:** Pau Benito, Mikel Isla-Jover, Pablo González-Castro, Pedro José Fernández Esparcia, Manuel Carpio, Iván Blay-Simón, Pablo Gutiérrez-Bedia, Maria J Lapastora, Beatriz Carratalá, Carlos Carazo-Casas

**Affiliations:** 1 Department of Preventive Medicine and Epidemiology, Clinical Institute of Medicine and Dermatology (ICMiD), Hospital Clínic de Barcelona, Rosselló, 138, ground floor, Barcelona, 08036, Spain, 34 932 27 54 00 ext 4046; 2 Department of Radiology, Hospital de Cruces, Barakaldo, Spain; 3 Department of Plastic and Reconstructive Surgery, Hospital Universitario Virgen del Rocío, Sevilla, Spain; 4 Department of Dermatology, Hospital Universitario Ramón y Cajal, Madrid, Spain; 5 Department of Endocrinology and Nutrition, Santa Lucía University General Hospital, Cartagena, Spain; 6 Department of Dermatology, Hospital Universitario Doctor Peset, Valencia, Spain; 7 Department of Neurology, Hospital Clínico San Carlos, Madrid, Spain; 8 Department of Intensive Care Medicine, Hospital Universitario 12 De Octubre, Madrid, Spain; 9 Innovation and Digital Projects Academic Department, Healthcademia, Madrid, Spain; 10 Department of Otolaryngology, Hospital Universitario Ramón y Cajal, Madrid, Spain

**Keywords:** accuracy, artificial intelligence, GPT-4o, large language models, medical education, medical examination, Médico Interno Residente, MIR 2024 examination, OpenAI o1

## Abstract

**Background:**

In recent years, generative artificial intelligence and large language models (LLMs) have rapidly advanced, offering significant potential to transform medical education. Several studies have evaluated the performance of chatbots on multiple-choice medical examinations.

**Objective:**

The study aims to assess the performance of two LLMs—GPT-4o and OpenAI o1—on the *Médico Interno Residente* (MIR) 2024 examination, the Spanish national medical test that determines eligibility for competitive medical specialist training positions.

**Methods:**

A total of 176 questions from the MIR 2024 examination were analyzed. Each question was presented individually to the chatbots to ensure independence and prevent memory retention bias. No additional prompts were introduced to minimize potential bias. For each LLM, response consistency under verification prompting was assessed by systematically asking, “Are you sure?” after each response. Accuracy was defined as the percentage of correct responses compared to the official answers provided by the Spanish Ministry of Health. It was assessed for GPT-4o, OpenAI o1, and, as a benchmark, for a consensus of medical specialists and for the average MIR candidate. Subanalyses included performance across different medical subjects, question difficulty (quintiles based on the percentage of examinees correctly answering each question), and question types (clinical cases vs theoretical questions; positive vs negative questions).

**Results:**

Overall accuracy was 89.8% (158/176) for GPT-4o and 90% (160/176) after verification prompting, 92.6% (163/176) for OpenAI o1 and 93.2% (164/176) after verification prompting, 94.3% (166/176) for the consensus of medical specialists, and 56.6% (100/176) for the average MIR candidate. Both LLMs and the consensus of medical specialists outperformed the average MIR candidate across all 20 medical subjects analyzed, with ≥80% LLMs’ accuracy in most domains. A performance gradient was observed: LLMs’ accuracy gradually declined as question difficulty increased. Slightly higher accuracy was observed for clinical cases compared to theoretical questions, as well as for positive questions compared to negative ones. Both models demonstrated high response consistency, with near-perfect agreement between initial responses and those after the verification prompting.

**Conclusions:**

These findings highlight the excellent performance of GPT-4o and OpenAI o1 on the MIR 2024 examination, demonstrating consistent accuracy across medical subjects and question types. The integration of LLMs into medical education presents promising opportunities and is likely to reshape how students prepare for licensing examinations and change our understanding of medical education. Further research should explore how the wording, language, prompting techniques, and image-based questions can influence LLMs’ accuracy, as well as evaluate the performance of emerging artificial intelligence models in similar assessments.

## Introduction

The continuous developments in recent years have positioned generative artificial intelligence (AI) as a topic of paramount public and scientific interest. These developments have resulted in the creation of gradually more sophisticated and efficient large language models (LLMs) [1].

Some of the most prominent examples are the increasingly advanced models derived from the GPT family, developed by OpenAI, which rely on deep neural networks [2]. In 2024, OpenAI released 2 highly promising models. Out of which one was GPT-4o, launched in May 2024, a multimodal model capable of processing text and image inputs and generating text outputs in real time. GPT-4o stands out in terms of rapid response times and efficiency [3]. The other one was OpenAI o1, launched in September 2024, a model only capable of processing and generating text, but trained with large-scale reinforcement learning (RL) to reason using chain of thought (CoT) and so possessing advanced reasoning capabilities, surpassing GPT-4o in competitive programming, mathematics, and scientific reasoning [4]. Despite the absence of comprehensive benchmark sets providing consistent evidence, it is reasonable to expect that the presence of sophisticated built-in reasoning-optimized mechanisms in LLMs such as OpenAI o1—trained with RL and CoT—diminishes the relative impact of complex prompting strategies. In such cases, simple zero-shot prompting may prove more effective, or at least equally effective, compared to few-shot and chain-of-thought prompting [4,5].

Chatbots for daily use have emerged to provide virtual assistance, personalized solutions, and task automation in a wide range of fields, including medical education, which has also embraced this trend [6]. Chatbots can be used as a learning aid to improve clinical skills at the undergraduate, residency training, and postgraduate levels of continuous medical education [7].

There are several previous experiences evaluating the performance of chatbots answering multiple-choice questions [7], including medical board examinations like the United States Medical Licensing Examination (USMLE) [8-10]. These assessments are helpful to understand the state of the art regarding LLMs’ performance in medical examinations. Furthermore, they pose questions about and provide insightful information to shape the content and characteristics of medical education and examinations.

In Spain, similarly to the USMLE, doctors are examined prior to the beginning of their specialized training. The test is called the *Médico Interno Residente* (MIR) examination and consists of a 4.5-hour-long examination that includes 210 multiple-choice questions with 4 options and only 1 correct answer. It is held on a yearly basis. The examination serves a double purpose. On the one hand, it is used to rank physicians to assess their eligibility for competitive medical specialist training positions. On the other hand, it ensures minimum requirements are met among candidates.

Evidence suggests a strong correlation between LLM performance across different input languages and the representativeness of each language in the pre-training corpus, a relationship that extends to retrieval-augmented generation LLMs [11,12]. To our knowledge, no study has evaluated GPT-4o’s performance on the Spanish MIR examination to date and, more importantly, no study has compared it to OpenAI o1, which owns enhanced reasoning capabilities that could potentially be an advantage when taking the MIR examination [4]. In a broader sense, there are few published studies that have evaluated the performance of LLMs when responding to medical questions in the Spanish language. Among them, the study by Guillen-Grima et al [13] reported a remarkable accuracy rate of 87% for GPT-4 on the 2022 MIR examination, while the study by Flores-Cohaila et al [14] showed an accuracy rate of 86% for GPT-4 on the 2022 Peruvian National Licensing Medical Examination. Other studies posing questions in Spanish from specific medical subjects have shown similar results, with performance rates of 83.7% for GPT-4o in anesthesiology [15] and 93.7% for GPT-4 in rheumatology [16].

The primary aim of this study is to assess the performance of GPT-4o and OpenAI o1 LLMs in passing the MIR examination and to compare them with the expert consensus from instructors of one of the largest MIR preparation academies (Academia AMIR) and the students’ mean results. The secondary aim of this study is to compare the performance of GPT-4o, OpenAI o1, expert consensus from AMIR instructors and students by medical subjects, question difficulty, and type of question (clinical case vs theoretical question and positive vs negative question) to better characterize AI chatbots’ capabilities, limitations, strengths, and weaknesses.

## Methods

### Study Design

This is a cross-sectional study assessing the performance of 2 LLMs (GPT-4o and OpenAI o1) in answering the MIR 2024 examination questions. The study compares the models’ performance against each other and against specifically trained humans (expert consensus from AMIR instructors and the mean results from MIR 2024 examination candidates).

### MIR 2024 Examination

In Spain, there are 46 medical specialties, each requiring a specific training period of 4 to 5 years as a resident physician (MIR) in an accredited health care institution. Access to each specialty training spot depends on the national ranking of candidates. The ranking is based on a final grade which comes from the MIR examination score (90%) and the candidate’s academic record (10%) [17,18].

A total of 15,114 candidates were admitted to the MIR 2024 examination, of whom 13,711 sat for the test, competing for 9007 specialty positions available in accredited healthcare institutions across Spain [18].

The examination, held on January 25, 2025, consisted of 200 multiple-choice questions, each with 4 answer choices, with only 1 correct option. The first 25 questions included linked images that were part of the questions’ content and could help or be necessary to answer them. Additionally, 10 reserve questions were included to replace any disputed questions due to typographical errors, ambiguous wording, or issues with multiple or missing correct answers. Participants were given 4 hours and 30 minutes to complete the examination [17,18].

As a safeguard against academic misconduct (ie, cheating), the MIR examination is administered in several different versions each year. Each version comprises an identical question set with a varied sequence. Version 0 is established as the canonical version for scoring and for the publication of the official answer key.

Version 0 of the MIR 2024 examination was obtained from the Spanish Ministry of Health website [19]. In the final analysis, the 25 questions requiring image interpretation were excluded since one of the LLMs evaluated (OpenAI o1) does not accept image inputs. This decision aimed to ensure fair comparability across all study arms, as providing images only to human participants and the other LLM (GPT-4o) would have introduced a systematic advantage for them. The 5 questions whose objections were accepted by the Spanish Ministry of Health were also excluded from the final analysis in order to approximate the real examination as closely as possible (the Spanish Ministry of Health accepted the objection for 6 questions but one of them was linked to an image, so it was already eliminated from the analysis). Nonetheless, the performance of the 4 study arms on these questions is also reported and discussed later in the study. Of the reserve questions, the 4 questions that did not replace any challenged question were also discarded from the analysis. Final analysis included 176 questions.

### Study Arms

This study compares the 4 distinct arms as follows: GPT-4o, OpenAI o1, expert consensus from AMIR instructors (henceforth “AMIR consensus”), and mean results of the MIR 2024 examination candidates (henceforth “students”).

#### GPT-4o

This model, OpenAI’s flagship in 2024, is characterized by rapid response times and efficiency. Although it is a multimodal model, in this study, only its text processing and generation capabilities were used. Image-linked questions were excluded to ensure comparability with OpenAI o1 model.

#### OpenAI o1

Designed to use large-scale RL and CoT reasoning, this model exhibits advanced reasoning capabilities, which could be particularly useful for answering questions in the MIR examination.

#### AMIR Consensus

Academia AMIR is a private, for-profit educational company operating in Spain, Portugal, and several Latin American countries, providing postgraduate health sciences training. Its core activities include preparing candidates for official examinations such as the MIR examination. The company employs faculty members who deliver these courses but are independent from the MIR examination process. They neither contribute to the examination’s development nor belong to any public organization involved in its preparation. A panel of these faculty—at least 2 per medical specialty—collaboratively answered the entire MIR 2024 examination after its administration and official content release by the Spanish Ministry of Health. Meeting in a hybrid format (combining in-person and remote participation), they established a consensus through discussion answering within 4.5 hours, mirroring the time allotted to candidates. The goal was to give candidates prompt performance feedback before the official answer key was published. Therefore, the faculty had unrestricted access to textbooks, scientific literature, and LLMs, though reported use was minimal and reserved for clarifying ambiguous questions prior to group consensus. This process produced an expert consensus answer list for the MIR 2024 examination.

#### Students

Candidates who took the examination were encouraged to submit their answer templates to EstimAMIR, an online platform developed by Academia AMIR. This platform provides students with a preliminary assessment of their results, initially using “AMIR consensus” answers and later incorporating the provisional and definitive correct answers published by the Spanish Ministry of Health. The platform also estimated each student’s ranking position based on the sample of candidates available. The mean results of the “students”, as well as the percentage of correct answers for each question, were obtained from this platform (based on 5066 answer templates submitted). All data were appropriately anonymized and aggregated.

### Data Collection

The 185 text-based questions from the MIR 2024 examination were collected and transcribed verbatim in Spanish into the dialogue interface of both GPT-4o and OpenAI o1. A ChatGPT Plus license was used to access the GPT-4o and OpenAI o1. The models were used with their default settings, with no modifications to parameters such as temperature or output variation. Each multiple-choice question was followed by the 4 possible answer choices (1, 2, 3, and 4), which were manually entered and separated by single spaces. No pretraining or standardized instructions were provided, adhering strictly to a zero-shot prompting approach to minimize potential bias. Henceforth, the results generated using this prompt will be designated as the first iteration results.

Questions were presented to the chatbots individually, with a new dialogue initiated for each question to ensure independence and prevent memory retention bias. To assess response consistency, chatbots were systematically challenged with the verification prompt “Are you sure?” after each answer, which served as a single CoT prompt. Hereafter, we referred to the results obtained with this prompt as the second iteration results. For GPT-4o, internet access was disabled during testing.

All responses were recorded in a spreadsheet. Once the definitive official answers were published by the Spanish Ministry of Health, any challenged or unused reserve questions were excluded from the final analysis.

### Main Endpoint and Additional Analysis

#### Overview

The primary endpoint was the percentage of correct answers per study arm. The definitive official answers published by the Spanish Ministry of Health served as the gold standard for determining accuracy within each study arm. The secondary endpoints included comparisons of study arm performance based on medical subjects, question difficulty, and question type (clinical case vs theoretical question and positive vs negative question). Categorization was conducted as described below.

#### Medical Subject

Questions were classified into the following categories such as gastroenterology and general surgery, endocrinology, infectious diseases and microbiology, miscellaneous and basic sciences, neurology and neurosurgery, cardiology and cardiovascular surgery, gynecology and obstetrics, orthopedic surgery, pediatrics, nephrology, respiratory medicine and thoracic surgery, rheumatology, hematology, psychiatry, immunology, urology, dermatology, ophthalmology, otorhinolaryngology, and statistics and epidemiology.

#### Question Difficulty

Difficulty was categorized based on the percentage of examinees who correctly answered each question, using data from EstimAMIR (very difficult: 0%‐20% correct responses; difficult: 21%‐40% correct responses; intermediate: 41%‐60% correct responses; easy: 61%‐80% correct responses; very easy: 81%‐100% correct responses).

#### Theoretical Question Versus Clinical Case

Questions were classified as theoretical (requiring a direct answer based exclusively on theoretical knowledge) and clinical case (presenting a clinical scenario from which the possible answers emerged).

#### Positive Versus Negative Questions

Questions were classified based on whether they asked for the correct answer (or the next appropriate step) or the incorrect answer (or the step that should not be taken).

### Statistical Analysis

Comparisons between study arms were performed using chi-squared tests or the Fisher exact test, where applicable. The Benjamini-Hochberg method was applied to statistically adjust for multiple comparisons. Differences between groups were considered statistically significant if *P*<.05. We assessed the consistency of responses from GPT-4o and OpenAI o1 to a verification prompt (“Are you sure?”). Consistency was measured using both the simple agreement percentage and the Cohen κ coefficient between the answers provided before and after the prompt. All statistical analyses were performed using *R* version 4.4.1 (R Foundation for Statistical Computing).

### Ethical Considerations

In the absence of a formal ethics committee at Academia AMIR, an ad hoc data ethical oversight panel approved a comprehensive data use protocol (internal reference: AMIR-ETH-2025-11-05-v1.0). At enrollment, all students provided data-use consent, acknowledging that their irreversibly anonymized and aggregated data could be used by Academia AMIR for statistical, commercial, educational, research, and product improvement purposes. The panel determined that the study qualified for exemption from institutional review board approval, as it involved secondary data that were aggregated, processed without human intervention, and contained no identifiable information, in accordance with principles of risk proportionality and data minimization. Participant privacy and confidentiality were safeguarded through irreversible anonymization and aggregation prior to investigator access, data minimization, role-based access controls, and encryption of data both in transit and at rest within corporate repositories. No participants received compensation for their participation in the study.

## Results

### Global Performance

**Figure 1.**
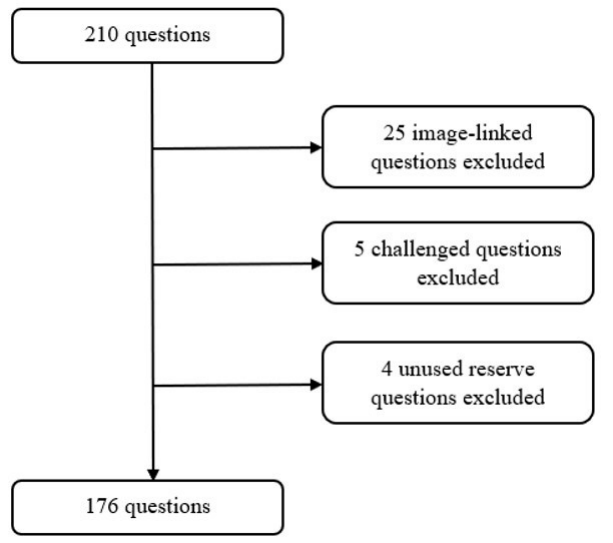
Flowchart of the exclusion criteria for the question selection process.

A flowchart of the exclusion criteria for the question selection process is shown in Figure 1.

Table 1 presents the accuracy of GPT-4o, OpenAI o1, the AMIR consensus, and students when taking the entire examination, excluding the discarded questions. GPT-4o achieved an accuracy of 89.8% (158/176) in the first iteration, which slightly increased to 90.9% (160/176) in the second iteration. Similarly, OpenAI o1’s accuracy was 92.6% (163/176) in the first iteration and improved to 93.2% (164/176) in the second iteration. The AMIR instructors’ consensus obtained the highest score among the study arms, with 94.3% (166/176). The mean score of the EstimAMIR-submitted templates was 56.6% (100/176).

**Table 1.** Global performance of GPT-4o (first and second iteration), OpenAI o1 (first and second iteration), AMIR consensus, and students.

	Absolute and relative performance	
	Correct answers, n (%)	Incorrect answers, n (%)
GPT-4o		
First iteration	158 (89.8)	18 (10.2)
Second iteration	160 (90.9)	16 (9.1)
OpenAI o1		
First iteration	163 (92.6)	13 (7.4)
Second iteration	164 (93.2)	12 (6.8)
AMIR consensus	166 (94.3)	10 (5.7)
Students	100 (56.6)	76 (43.4)

**Figure 2.**
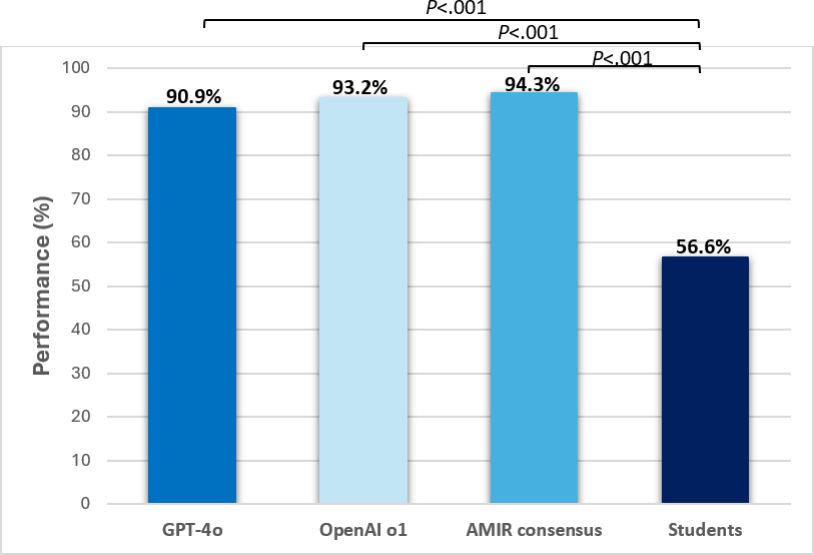
Global performance of GPT-4o (2nd iteration), OpenAI o1 (2nd iteration), AMIR consensus, and students.

Figure 2 compares the accuracy of GPT-4o and OpenAI o1 in their second iterations, along with the AMIR consensus and students. GPT-4o, OpenAI o1, and the AMIR consensus achieved significantly higher accuracy scores than the average student (in all cases *P*<.001); however, differences between these 3 arms did not reach statistical significance (*P*=.22 for GPT-4o vs OpenAI o1; *P*=.07 for GPT-4o vs AMIR consensus; *P*=.75 for OpenAI o1 vs AMIR consensus).

From the final analysis, 5 challenged questions were excluded. On these items, GPT-4o achieved an accuracy of 100% (5/5) across both iterations. OpenAI o1 scored 80% (4/5) in both iterations, without modifying any of its responses. The AMIR consensus achieved an accuracy of 40% (2/5). When restricted to these 5 questions, the students’ mean score was 31.3% (SD 16.7%).

### Medical Subjects

**Figure 3.**
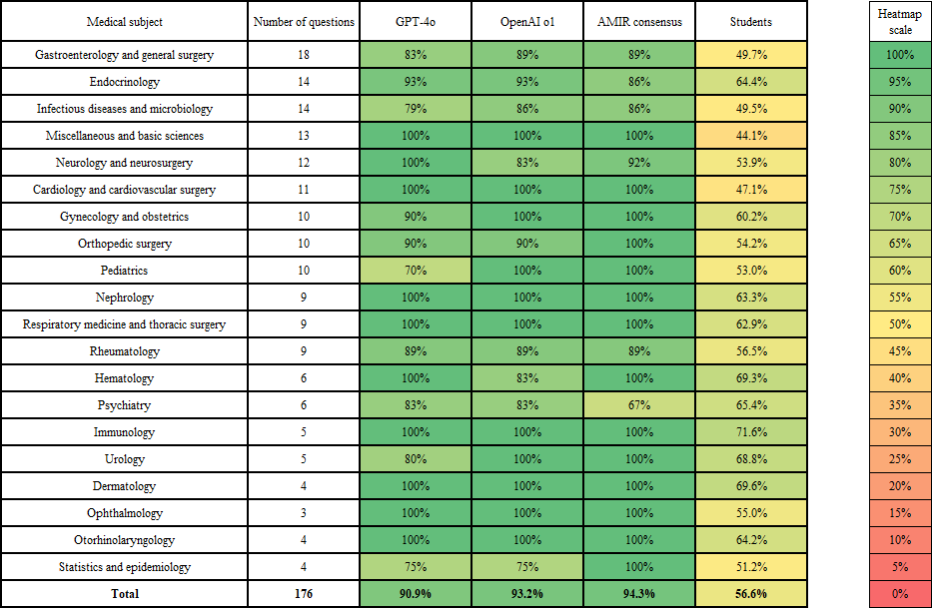
Heatmap comparing the performance of generative pre-trained transformer 4o (2nd iteration), OpenAI o1 (2nd iteration), and AMIR consensus and students by medical subject.

The heatmap in Figure 3 presents a comparative analysis of study arm performance across different medical subjects. Both LLMs (GPT-4o and OpenAI o1, in their second iterations) and the AMIR consensus outperformed the average student in all 20 medical subjects analyzed.

GPT-4o achieved an accuracy below 80% in only 3 subjects: infectious diseases and microbiology (11/14, 79%), pediatrics (7/10, 70%), and statistics and epidemiology (3/4, 75%). OpenAI o1 fell below 80% accuracy only in statistics and epidemiology, with a single error (3/4, 75%). The AMIR consensus exhibited an accuracy lower than 80% in psychiatry (4/6, 67%). Average student performance, based on 5066 EstimAMIR-submitted templates, ranged from 44.1% in miscellaneous and basic sciences to 71.6% in immunology.

### Question Difficulty

**Figure 4.**
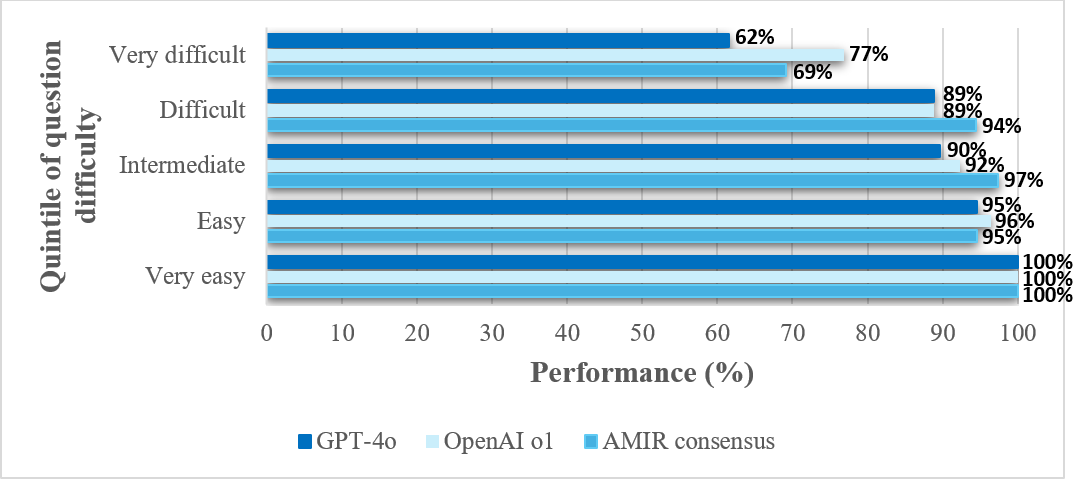
Performance of GPT-4o (2nd iteration), OpenAI o1 (2nd iteration), and AMIR consensus by quintiles of question difficulty defined by students’ performance.

As shown in Table 2 and Figure 4 the performance of GPT-4o, OpenAI o1, and the AMIR consensus across quintiles of question difficulty, defined based on student performance. A statistically significant gradient is observed among these 3 study arms, with accuracy decreasing as question difficulty increases (crude *P* values: GPT-4o, *P*=.003; OpenAI o1, *P*=.04; AMIR consensus, *P*=.008; Benjamini-Hochberg adjusted *P* values: GPT-4o, *P*=.03; OpenAI o1, *P*=.10—the only case not reaching statistical significance; AMIR consensus, *P*=.03). The decline in performance is particularly pronounced in the highest difficulty quintile. Differences between study arms within each difficulty quintile do not reach statistical significance.

**Table 2.** Absolute and relative performance of GPT-4o (second iteration), OpenAI o1 (second iteration), and AMIR consensus by quintiles of question difficulty defined by students’ performance.

Question difficulty	Number of questions	GPT-4o, n (%)	OpenAI o1, n (%)	AMIR consensus, n (%)
Very easy	32	32 (100)	32 (100)	32 (100)
Easy	56	53 (95)	54 (96)	53 (95)
Intermediate	39	35 (90)	36 (92)	38 (97)
Difficult	36	32 (89)	32 (89)	34 (94)
Very difficult	13	8 (62)	10 (77)	9 (69)
Total	176	160 (90.9)	164 (93.2)	166 (94.3)

### Clinical Cases versus Theoretical Questions

As shown in Table 3 the performance of GPT-4o, OpenAI o1, the AMIR consensus, and students when answering clinical cases versus theoretical questions. Overall, a slightly higher accuracy was observed for clinical cases compared to theoretical questions, although these differences do not reach statistical significance in any study arm. Statistically significant differences between study arms both for clinical cases and theoretical questions were observed only when the students' arm was included in the analysis.

**Table 3.** Performance of GPT-4o (second iteration), OpenAI o1 (second iteration), AMIR consensus, and students by questions being clinical cases or theoretical questions.

	Clinical cases (n=105), n (%)	Theoretical questions (n=71), n (%)	*P* value
GPT-4o	98 (93.3)	62 (87)	.55
OpenAI o1	99 (94.2)	65 (92)	.68
AMIR consensus	99 (94.2)	67 (94)	.99
Students	62 (59)	38 (54)	.68

### Positive Versus Negative Questions

Table 4 shows the performance of GPT-4o, OpenAI o1, the AMIR consensus, and students when answering positive versus negative questions. Overall, accuracy was higher for positive questions than for negative ones, with the difference reaching statistical significance only for GPT-4o (*P*=.01). Statistically significant differences between study arms for both positive and negative questions are observed only when the students' arm is included in the analysis.

**Table 4.** Performance of GPT-4o (second iteration), OpenAI o1 (second iteration), AMIR consensus, and students by questions being positive or negative.

	Positive questions (n=140), n (%)	Negative questions (n=36), n (%)	*P* value
GPT-4o	132 (94.2)	28 (78)	.01
OpenAI o1	132 (94.2)	32 (89)	.40
AMIR consensus	132 (94.2)	34 (94)	1
Students	82 (58.5)	18 (49)	.55

### Response Consistency

Response consistency was assessed using the simple agreement percentage between the first and second iterations of GPT-4o (172/176, 97.7%) and OpenAI o1 (170/176, 96.6%). The Cohen κ coefficient was 0.97 for GPT-4o and 0.95 for OpenAI o1, indicating almost perfect agreement (*P*<.001 in both cases).

When analyzing individually the questions in which there was no concordance between the first and second iterations, it was observed that, for GPT-4o, 4 initially incorrect responses were modified: in 2 cases, the second response was also incorrect, while in the other 2 cases, the second response became correct. For OpenAI o1, 5 initially incorrect responses were modified: in 3 cases, the second response was again incorrect, and in 2 cases, the second response became correct. In addition, 1 initially correct response was modified, with the second response becoming incorrect.

## Discussion

### Principal Results

This study highlights the exceptional performance of both LLMs analyzed—GPT-4o and OpenAI o1—on the MIR 2024 examination. Both models achieved or exceeded a 90% accuracy rate, significantly outperforming the average human candidate as well as the top 10% of examinees [18]. The expert consensus from AMIR instructors yielded even higher accuracy. Although this result should be interpreted in the context of unrestricted access to textbooks, scientific literature, and AI tools such as GPT, the reported use of these resources was minimal and reserved for clarifying ambiguous questions, never substituting for the group discussion and consensus process for each item. Results from the expert consensus suggest the added value of human expertise when synergistically combined with AI capabilities.

The challenged questions were excluded from the final analysis to remain faithful to the actual examination. Upon examination, these proved to be difficult items (on average, candidates answered them correctly in 31.3% of cases, compared with 56.6% for the other questions), which the LLMs managed more accurately than the human experts (5/5 for GPT-4o, 4/5 for OpenAI o1, and 2/5 for AMIR instructors).

These results were consistent across the different medical subjects analyzed. Interestingly, when question difficulty was assessed based on human performance, a similar trend was observed in the LLMs, with accuracy decreasing as question difficulty increased. Additionally, a slightly higher accuracy was observed for clinical cases compared to theoretical questions, as well as for positive questions compared to negative ones. This resemblance to human reasoning and performance could be rooted in the input used to train LLMs.

Both GPT-4o and OpenAI o1 demonstrated great consistency in their answers, with statistically significant near-perfect agreement between the first and second iterations. Furthermore, it is particularly interesting to note that, of the 10 responses that were altered between the first and second iterations (4 for GPT-4o and 6 for OpenAI o1), 9 were initially incorrect (with 4 of these changing to a correct response), and only 1 initially correct response was changed to an incorrect one. It is remarkable in favor of these LLMs that, considering their exceptional accuracy in the first iteration, the few changes occurring in the second iteration almost exclusively involved some of the few initially incorrect responses.

### Comparison With Prior Work

Several previous studies have evaluated the performance of GPT-3.5 and GPT-4 across different medical disciplines, as well as on national medical board examinations [7]. For instance, Gilson et al assessed ChatGPT’s performance on various sets of USMLE step 1 and step 2 questions, reporting an accuracy range of 42% to 64% [8], which aligns with another study that found a 56% accuracy rate on a set of USMLE step 1-style questions [9]. Knoedler et al examined ChatGPT-3.5 and ChatGPT-4 on USMLE step 3 questions, reporting 57% accuracy for GPT-3.5 and 85% for GPT-4 [10]. Takagi et al evaluated these models on the Japanese Medical Licensing Examination, finding 51% accuracy for GPT-3.5 and 80% for GPT-4 [20]. Meyer et al conducted a similar study on the written German medical licensing examination, with accuracy rates of 58% for GPT-3.5 and 85% for GPT-4 [21]. A study by Prazeres [22] on the Portuguese national examination for access to specialized training reported 54% accuracy for GPT-3.5 turbo and 65% for GPT-4o mini. Guillen-Grima et al [13] published the perhaps most comparable study, as they compared GPT-3.5 and GPT-4 on the MIR 2022 examination. Accuracy rates were 63% for GPT-3.5 and 87% for GPT-4. Interestingly, our results show the highest accuracy for LLMs among all the aforementioned studies, almost matching the consensus from expert human instructors. This may be a result of the gradual development of LLMs with time. It poses relevant questions regarding how medical education and examinations should be shaped in the future, both in terms of content and the skills that are underscored. As stated in a previous editorial, these results make it important to consider the necessity of more emphasis on soft skills and critical thinking rather than plain memorization [23].

### Strengths

This study offers new insights into the accuracy of GPT-4o and OpenAI o1 in a national medical specialty access examination. To date, the only comparable research published in an indexed journal that we have identified is a recent study by Liu et al [24], which evaluated GPT-4o’s performance on the Japanese national medical examination, reporting an accuracy of 89%.

Moreover, this study reinforces the trend that each newly developed LLM exhibits improved accuracy compared to its predecessors. Additionally, our secondary analysis proved that the accuracy of both GPT-4o and OpenAI o1 aligned with difficulty levels of questions based on human candidates’ performance.

### Limitations

This study has several limitations. First, increasing the sample size—that is, including a larger number of questions in the analysis—would have provided a more robust insight into the different subanalyses performed. For instance, it would have allowed us to investigate whether performance differences of LLMs compared to human experts—such as those suggested in unique medical subjects like psychiatry—are truly meaningful or just the results of random variation. Second, additional secondary analyses could have been of interest, such as examining the relationship between the number of words or characters in each question and the performance of LLMs; the influence of specific expressions or wording styles on model accuracy; the impact of different languages on performance; and the effect of alternative prompting formulas on accuracy. Third, although image-based questions are part of the MIR examination, they were not included in this study because OpenAI o1 does not support image inputs, and fair comparability between LLMs was prioritized. This decision reduces methodological bias—LLMs are not artificially penalized for lacking multimodal capabilities—and increases the internal validity of between-arms comparisons. However, it may reduce the representativeness of the performance evaluation and limit the generalizability of our findings to the actual test setting, where visual interpretation is an integral component of clinical reasoning. Previous studies suggest LLMs may exhibit reasonable performance on image-based questions even without access to the image itself [25]. Fourth, the AMIR consensus may not represent a pure benchmark of human expert knowledge, as experts had access to textbooks and generative AI. However, the faculty use of these resources was minimal and strictly advisory, with all final answers determined by expert discussion and consensus, indicating that the potential for significant bias was low. Fifth, the field of LLMs is continuously evolving, and several new models have been released in recent months that were not analyzed in this study, including GPT-4.5 by OpenAI [26], DeepSeek-R1 by DeepSeek-AI [27], Qwen 2.5 by Alibaba [28], LlaMa 3.2 by Meta AI [29], and Claude 3.7 Sonnet by Anthropic [30], among others. Sixth, student results were self-reported, which could be a source of bias. Finally, caution should be exercised when generalizing LLM accuracy on the MIR examination to other national medical licensing examinations or to different fields and tasks within medical education.

### Conclusions

This study highlights the excellent performance of the two analyzed LLMs—GPT-4o and OpenAI o1—on the MIR 2024 examination, demonstrating strong consistency across different medical subjects and types of questions, as well as between first and second iterations.

The integration of LLMs into medical education is promising and likely to revolutionize the field and change our understanding of medical education. Further research is needed to explore how wording, language, prompting techniques, and image-based questions influence LLM accuracy in national medical licensing examinations, as well as to assess the performance of other emerging models. More research is also needed to better understand the potential usefulness of these tools as learning assistants in broader educational contexts.

## ABBREVIATIONS

AI: artificial intelligence
CoT: chain of thought
LLM: large language model
MIR: Médico Interno Residente
RL: reinforcement learning
USMLE: United States medical licensing examination
